# Single-cell multi-omics analysis of lineage development and spatial organization in the human fetal cerebellum

**DOI:** 10.1038/s41421-024-00656-1

**Published:** 2024-02-26

**Authors:** Fuqiang Yang, Ziqi Zhao, Dan Zhang, Yu Xiong, Xinran Dong, Yuchen Wang, Min Yang, Taotao Pan, Chuanyu Liu, Kaiyi Liu, Yifeng Lin, Yongjie Liu, Qiang Tu, Yashan Dang, Mingyang Xia, Da Mi, Wenhao Zhou, Zhiheng Xu

**Affiliations:** 1 State Key Laboratory of Molecular Developmental Biology, Institute of Genetics and Developmental Biology, University of Chinese Academy of Sciences, Chinese Academy of Sciences, Beijing, China; 2 Shanghai Key Laboratory of Female Reproductive Endocrine-Related Diseases, Obstetrics and Gynecology Hospital of Fudan University, Shanghai, China; 3 Center for Molecular Medicine, Children’s Hospital of Fudan University, Shanghai, China; 4 Department of Neonatology, Obstetrics and Gynecology Hospital of Fudan University, Shanghai, China; 5 BGI-Beijing, Beijing, China; 6 BGI-Shenzhen, Shenzhen, China; 7 Key Laboratory of Birth Defects, Children’s Hospital of Fudan University, Shanghai, China; 8 State Key Laboratory of Membrane Biology, Tsinghua-Peking Center for Life Sciences, IDG/McGovern Institute for Brain Research, School of Life Sciences, Tsinghua University, Beijing, China; 9 Guangzhou Women and Children’s Medical Center, Guangzhou Medical University, Guangzhou, Guangdong, China

**Keywords:** Developmental biology, Bioinformatics

## Abstract

Human cerebellum encompasses numerous neurons, exhibiting a distinct developmental paradigm from cerebrum. Here we conducted scRNA-seq, scATAC-seq and spatial transcriptomic analyses of fetal samples from gestational week (GW) 13 to 18 to explore the emergence of cellular diversity and developmental programs in the developing human cerebellum. We identified transitory granule cell progenitors that are conserved across species. Special patterns in both granule cells and Purkinje cells were dissected multidimensionally. Species-specific gene expression patterns of cerebellar lobes were characterized and we found that *PARM1* exhibited inconsistent distribution in human and mouse granule cells. A novel cluster of potential neuroepithelium at the rhombic lip was identified. We also resolved various subtypes of Purkinje cells and unipolar brush cells and revealed gene regulatory networks controlling their diversification. Therefore, our study offers a valuable multi-omics landscape of human fetal cerebellum and advances our understanding of development and spatial organization of human cerebellum.

## Introduction

The complex array of neuron types in the cerebellum occupies >80% of total neurons in the human brain^1^. Integrated neural networks in the different cerebellar regions coordinate various functions including motor, cognition, emotion and language^2–5^. Impairment of cerebellar circuits would result in dysmetria of thought or movement^6^. Reduced grey matter in the cerebellar cortex was also observed in autism spectrum symptom or attention disorder^7^. The architecture of the cerebellar cortex is relatively conserved throughout the evolution^8^. Almost all kinds of vertebrates have three layers in the mature cerebellum, namely molecular layer (ML), Purkinje cell layer (PCL) and granule cell layer (GCL)^8^. Above the white matter, the GCL contains intensive granule cells (GCs), which is covered by a layer of Purkinje cells (PKCs)^9^. The ML consists of the dendritic trees of PKCs and the parallel fibers of GCs^9^. Various kinds of interneurons interweave within different layers and harness the signal transduction in the cerebellum^10^.

**Fig. 1:**
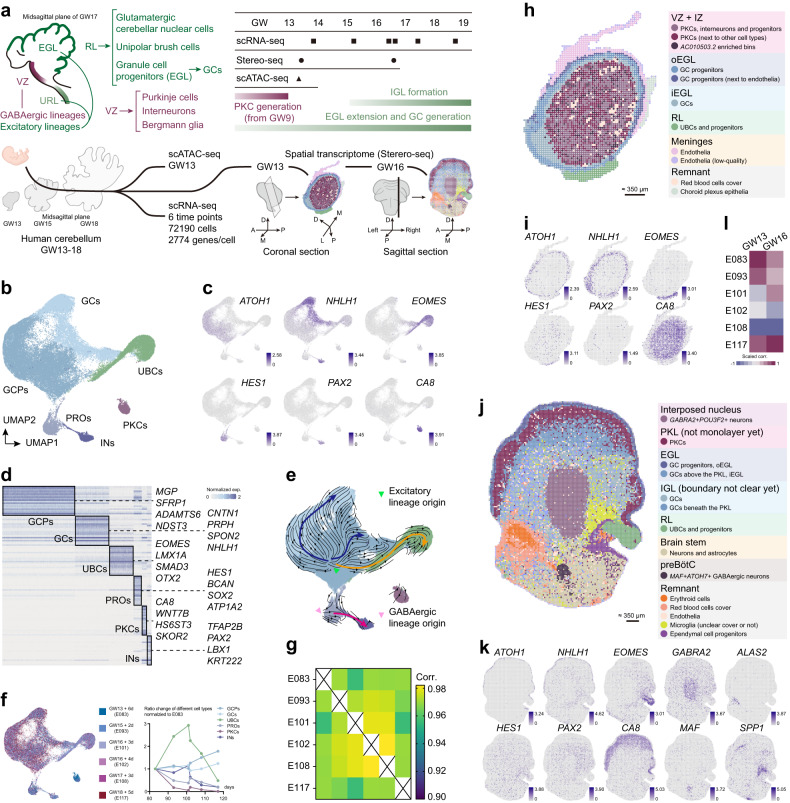
Multi-omics profiling of human cerebellar development. **a** Schematic overview of the development of major cell types in the human cerebellum (top left) and time points of fetal samples and corresponding biological events (top right). Schematic overview of the multi-omic analyses (bottom). **b** Visualization of integrated human scRNA-seq data with cell identities color coded. GCPs, GC progenitors. PROs, progenitors. INs, interneurons. **c** Gene expression patterns of marker genes in integrated scRNA-seq. Grey represents low levels and navy blue represents high levels. **d** Heatmap showing the marker genes of different cell types. **e** Gene velocity flow map visualized on UMAP embedding of integrated data. Most naïve sites are labeled by green and pink arrowheads. Streamlines represent the RNA velocity predicting the future transcriptional dynamic state of cells as concluded by blue, yellow, and pink curves. **f** Contribution of human samples and major cell types from different time points in scRNA-seq data (left). Line graph showing the ratio change of different cell types (right). **g** Correlations between scRNA-seq data from different time points. **h**–**k** Spatial transcriptomic data of coronal slice at GW13 (**h**) and sagittal slice at GW16 (**j**) with region identities color coded. Spatial gene expression patterns of marker genes of different cell types at GW13 (**i**) and GW16 (**k**). **l** Correlations between spatial transcriptomic data and scRNA-seq data from different time points.

During the development, the majority of neurons in the cerebellum are derived from two germinal centers, the ventricular zone (VZ) and upper rhombic lip (URL)^11^ (Fig. 1a). GABAergic lineages including PKCs and various interneurons are generated from the VZ, as well as Bergmann glia^11^. PKCs are generated first, then radially migrate and are organized into the PCL^11^. Excitatory lineages including deep cerebellar nucleus neurons, GCs and unipolar brush cells (UBCs) are derived from the URL^12^. While the UBC progenitors settle in the URL, proliferative GC progenitors migrate tangentially to cover the surface of the cerebellum, forming the external granular layer (EGL) as the secondary germinal zone^12–14^. Since postmitotic GCs form the inner EGL (iEGL) after inward migration, the separation between GC progenitors in the outer EGL (oEGL) and GCs occurs^14^. GCs will continue to migrate and pass through PKCs to form the internal granular layer (IGL) which finally develops into the GCL^14^.

Several studies have applied single cell technologies to investigate the details of mouse cerebellar development from embryonic to postnatal stages^15–24^. However, our understanding of human cerebellar development is relatively limited^25–27^. Here, we adopted single-cell multi-omics approaches, including single-cell RNA sequencing (scRNA-seq), single-cell sequencing assay for transposase-accessible chromatin (scATAC-seq) and spatial transcriptomics, to delineate the developmental landscape of human cerebellum at high spatial-temporal resolution. We identified different GC progenitors which are conserved across species and characterized the species-specific spatial pattern of gene expression within GC lineages under different dimensions. Multidimensional features of PKC subtypes were identified. Gene regulatory networks (GRNs) involved in the diversification of different cell types were generated to facilitate our understanding of cerebellar lineage development.

## Results

### High-quality multi-omics profiling of the developing human cerebellum

We applied scRNA-seq, scATAC-seq and spatial transcriptomics approaches to obtain high-quality multi-omics profiles of the developing human cerebellum. scRNA-seq covering human GW13–GW18 samples, spatial transcriptomic analysis (Stereo-seq) of GW13 and GW16 samples, and scATAC-seq of GW13 sample were performed (Fig. 1a). A series of published mouse scRNA-seq data were also re-analyzed for the cross-species comparison^15,19^.

Transcriptional profiles of 72,190 high-quality human cells were obtained with an average capture of 2774 genes/cell (Supplementary Table S1). Microglia, endothelial cells and red blood cells were removed in each dataset. Using Seurat package^28^, scRNA-seq data of the remaining 62,247 cells were integrated and visualized under the embedding of uniform manifold approximation and projection (UMAP) (Fig. 1b). GABAergic lineages such as interneurons (*PAX2*^+^), PKCs (*CA8*^+^*WNT7B*^*+*^) and excitatory lineages including UBCs (*EOMES*^+^*LMX1A*^*+*^), GC progenitors (*ATOH1*^+^*LHX9*^*–*^) and postmitotic GCs (*NHLH1*^*+*^*LHX9*^*–*^*TBR1*^*–*^) were identified (Fig. 1c; Supplementary Fig. S1a). *HES1*^*+*^ progenitors and glia cells which are mainly derived from the VZ were also classified (Fig. 1b, c). More cell type-specific markers were explored using the COSG method^29^ (Fig. 1d; Supplementary Table S2).

Previous studies have found that *ATOH1* is highly expressed in URL-derived excitatory progenitors including GC progenitors in the EGL, while *PTF1A* is enriched in progenitors emerging from the VZ^8^. The RNA velocity analysis identified the consistent naïve progenitors as reported (Fig. 1e). Co-expression of G2/M phase markers (e.g., *TOP2A*) and *ATOH1* was observed, while the co-expression of *TOP2A* and *PTF1A* was barely detected (Fig. 1c; Supplementary Fig. S1a). It is likely due to the small proportion of *PTF1A*^*+*^ progenitors compared with excitatory progenitors. Furthermore, the highest abundance of PKCs was observed in GW13, which decreased sharply later (Fig. 1f; Supplementary Fig. S1b, c and Table S3). Plausibly, PKC production is already completed around GW13^30^ (Fig. 1a) while the EGL is rapidly proliferating, producing large numbers of GCs that quickly outnumber PKCs. The ratio of UBCs was found to peak around GW16 (Fig. 1f), consistent with a very recent study^26^. Each dataset also correlated well with data from adjacent time points (Fig. 1g). These results suggested that our scRNA-seq data generally captured the characteristics of human cerebellar development.

Our spatial transcriptomics data were analyzed at bin 50 resolution (50 × 50 DNA nanoballs (DNBs), diameter is ~35 μm, see Materials and methods). 3609 bins with an average of 2481 genes/bin and 15,880 bins with an average of 1890 genes/bin were profiled in samples collected at GW13 and GW16, respectively (Fig. 1h–k; Supplementary Table S4). Correlation between scRNA-seq and spatial transcriptomic data were calculated based on pseudobulk of each dataset (Fig. 1l). Unsupervised clustering based on binned data suggested a multi-layered annular distribution of different cell types in GW13 spatial transcriptomic data, correlated with the oEGL, iEGL, IZ and VZ regions, respectively (Fig. 1h; Supplementary Fig. S1d). Clear separation of the rhombic lip (RL), EGL, and Purkinje cell layer (PKL) was also detected in GW16 spatial transcriptomic data, as well as interposed nucleus and nucleus embedded in the brainstem (Fig. 1j; Supplementary Fig. S1d). By using ArchR package^31^, the accessibility of each gene in GW13 scATAC-seq dataset was scored, followed by cell type clustering analysis (Supplementary Fig. S1e–g). We found a comparable resolution of cell type classification by using scATAC-seq dataset solely or integration of scATAC-seq and scRNA-seq datasets (Supplementary Fig. S1e).

### Developmental trajectory and transient amplifying progenitors in GC lineages

**Fig. 2:**
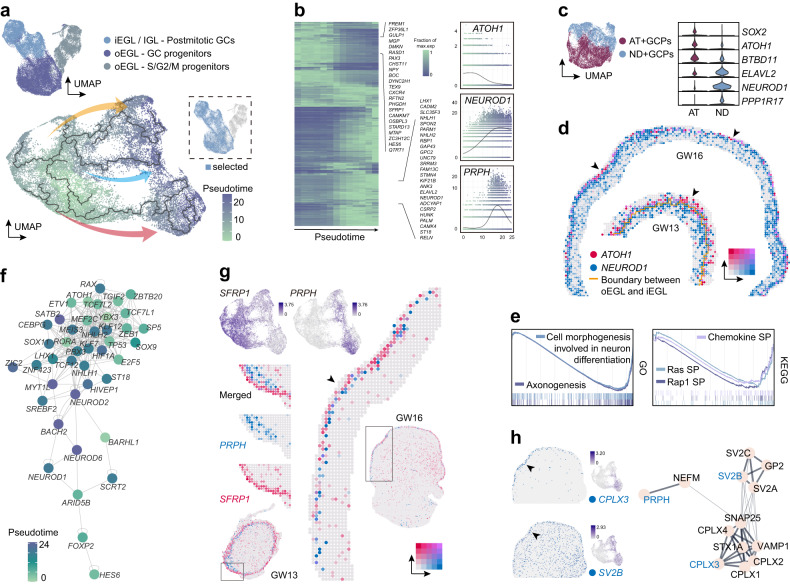
Trajectory inference and EGL progenitor subtypes in the GC lineage. **a** Visualization of GC lineages in different phases of cell cycle (top). Pseudotime trajectory visualized on UMAP embedding of G1 phase GC progenitors and postmitotic GCs (bottom). Blue, orange and pink arrows mark three potential sublineages suggested by the trajectory. **b** Altered expression patterns of different genes along the pseudotime. An overview (left) and the detailed patterns of several genes (right). **c** Visualization of the AT+GCP and ND+GCP (left) and violin plot showing the expression patterns of their marker genes (right). **d** Spatial gene expression patterns showing the separated expressions of *ATOH1* and *NEUROD1* in both GW13 and GW16 EGL in human cerebellum. **e** Enrichment plots of GO and KEGG terms in AT+GCP compared with ND+GCP. **f** GRNs of GC development. **g** Gene expression patterns of *SFRP1* and *PRPH* in scRNA-seq and spatial RNA-seq data. **h** Spatial gene expression patterns of *CPLX3* and *SV2B* at GW16 with arrowheads highlighting the enrichment in the EGL (left). Protein interaction network according to STRING (right).

GCs account for the majority of cerebellar excitatory neurons featured by the expression of *NRN1*^1,32^. To investigate the development of GCs, we first performed cell cycle scoring of *NRN1*^+^*EOMES*^*–*^ GC lineage cells. Highly proliferative *SFRP1*^+^*ATOH1*^+^ GC progenitors in G1 or S/G2/M phase, as well as *NHLH1*^*+*^*STMN2*^*+*^ postmitotic GCs could be distinguished (Fig. 2a; Supplementary Fig. S2a, b). The gene ontology (GO) enrichment analysis confirmed the high activity of axon development in postmitotic GCs compared with progenitors (Supplementary Fig. S2c), suggesting the ongoing of neural differentiation.

Pseudotime trajectory of GC lineage was then constructed based on G1 phase cells by using Monocle3 package^33^ (Fig. 2a), with the most naïve point calculated by using CytoTRACE and StemSC packages^34,35^ (Supplementary Fig. S2d). We noticed that the elevation of *NEUROD1* expression tends to be earlier than other markers of postmitotic GCs along the trajectory (Fig. 2b). Co-expression of *NEUROD1* and S/G2/M phase markers was also found, which indicated the proliferative feature of these cells (Supplementary Fig. S2a). Further classification distinguished two groups of GC progenitors, which were labeled as *ATOH1*^+^ GC progenitors (AT+GCPs) and *NEUROD1*^+^ GC progenitors (ND+GCPs). While the ND+GCPs expressed higher level of *NEUROD1* and *ELAVL2*, almost no *SOX2* was detected in ND+GCPs compared with AT+GCPs (Fig. 2c). Similarly, distinction of these two GC progenitors could be found in mouse (Supplementary Fig. S2e, f). As reviewed recently, the ND+GCPs resemble the transient amplifying progenitors in mouse, which reside beneath the AT+GCP^36^. Such an inside-outside separation could be observed especially in our GW13 spatial transcriptomic data, in which clear boundary of iEGL and oEGL was detected (Fig. 2d). Gene set enrichment analysis (GSEA) indicated the activation of chemokines, Ras or Rap1 signaling pathways regarding the morphological changes during neural differentiation in both human and mouse ND+GCPs, suggesting the conserved regulatory pathways across species (Fig. 2e; Supplementary Fig. S2g).

Furthermore, transcription factors enriched in either progenitors or postmitotic cells were selected for the GRN analysis through the integration of scRNA-seq and scATAC-seq data. We leveraged the inferred developmental trajectories and the GRNs to identify key genes which may regulate the cell state transitions during the differentiation of GCs (Fig. 2f). Apart from *ATOH1*, transcription factors like *YBX3* and *MEF2C* were discovered to be potentially involved in the development of GCs (Fig. 2f). MEF2C could be detected in the GCs of human embryonic cerebellum while its function remains unclear^37^.

Interestingly, we found a marker *PRPH* in postmitotic GCs based on the pseudotime trajectory of GC lineages. *PRPH* showed an almost complementary expression pattern with *SFRP1* but not detectable in the most matured GCs (Fig. 2g). Consistent with scRNA-seq data, non-overlapping expression patterns of *SFRP1* and *PRPH* was visualized in both of our spatial transcriptomic data (Fig. 2g). The expression of *PRPH* was almost constrained in the iEGL and hardly detected in the migrating and IGL GCs (Fig. 2g). *PRPH* could be used as an iEGL marker which is specifically expressed in the postmitotic GCs. Genes with similar expression patterns were further explored. We found that *CPLX3* was uniquely detected in anterior iEGL while *SV2B* was enriched in iEGL with less specificity (Fig. 2h). *PRPH* encodes the neuronal intermediate filament protein peripherin whose mutation could result in disruption of neurofilament network assembly^38^. Both CPLX3 and SV2B were reported to participate in the regulation of neurotransmitter secretion^39,40^. These proteins seemed to be involved in a protein interaction network according to the STRING database^41^ (Fig. 2h). We speculated that this protein interaction network was involved in the regulation of GC migration in a region-specific way.

### GC sublineages and their multidimensional spatial distribution

**Fig. 3:**
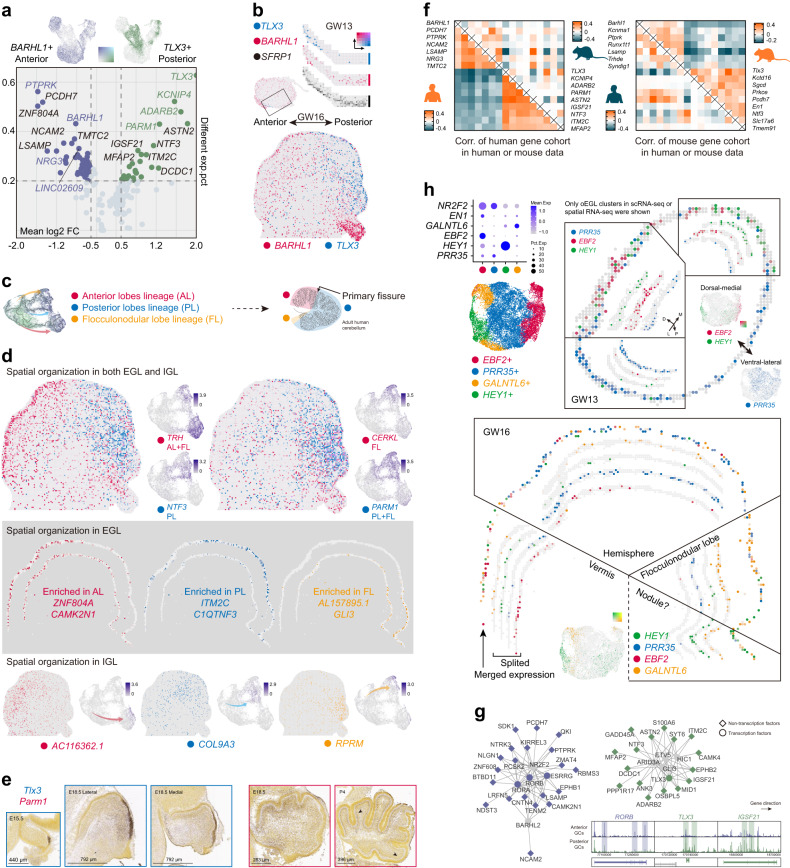
Spatial separation of GC lineages and characterization of sublineages. **a** Gene expression patterns of *BARHL1* and *TLX3* in GC lineages (top). Volcano plot showing the complementarily expressed gene cohorts (bottom). Symbols of almost evenly expressed genes in each subtype are colored blue (anterior) or green (posterior). **b** Spatial gene expression patterns of *BARHL1* in anterior regions and *TLX3* in posterior regions in both GW13 (top) and GW16 (bottom). *SFRP1* labeled GC progenitors. **c** Schematic overview of three GC sublineages corresponding to three lobes in the human cerebellum. **d** Spatial organizations of GC sublineages in EGL and IGL. Genes detected in both EGL and IGL (top). Genes (the first gene corresponds to the outside one) enriched in EGL of each lobe (middle). Genes enriched in IGL of each lobe (bottom). **e** In situ hybridization results of *Tlx3* and *Parm1* in mouse at E15.5 and E18.5 adopted from Allen Brain (https://developingmouse.brain-map.org). Arrowheads indicated the detection of *Parm1* in anterior but not in posterior lobes of mouse. **f** Correlation of human (left) or mouse (right) gene cohorts in human and mouse datasets showing species-specific spatial gene expression patterns. In each panel the bottom-left parts are human datasets. Only G1 phase GC lineage cells were calculated. **g** GRNs showing the potential regulatory programs of anterior (blue) or posterior (green) specific genes (top left) and visualization of accessible regions in different genes (bottom right). **h** Visualization of four subtypes of GC progenitor cells in scRNA-seq data and the expression of their marker genes (top left). Spatial distributions of *PRR35*, *EBF2* and *HEY1* in oEGL of GW13 (top right). Spatial distributions of *HEY1*, *PRR35*, *EBF2* and *GALNTL6* in EGL of GW16 (bottom).

Neural circuit analyses have unveiled the functional preference in different cerebellar regions, such as anterior lobes for sensorimotor functions, and lateral posterior lobes for cognition^2^. Since *CPLX3* is specifically expressed in the anterior region (Fig. 2h), three sublineages of GCs labeled in the pseudotime trajectory may also resemble the GCs organized in different regions (Fig. 2a). Further analysis unveiled mutually exclusively expressed gene cohorts represented by *BARHL1* and *TLX3* in both GC progenitors and postmitotic GCs (Fig. 3a; Supplementary Fig. S3a and Table S5). The anterior-posterior (A-P) distribution of *BARHL1* and *TLX3* was clearly observed especially in the sagittal section of GW16 (Fig. 3b). Three sublineages recognized in the pseudotime trajectory corresponding to the anterior lobes, posterior lobes and flocculonodular lobe, with both flocculonodular and posterior lobes being *TLX3*^+^ in human (Fig. 3c, d). Similar gene expression patterns could be found in both scRNA-seq and spatial transcriptomic data, such as *CERKL* enriched in the flocculonodular lobe, *NTF3* in the posterior lobes and *TRH* in both flocculonodular and anterior lobes (Fig. 3d). Region-specific gene expression in either EGL progenitors or IGL postmitotic GCs could be observed (Fig. 3d).

Both a previous report and in situ hybridization data from Allen Brain showed that mouse *Tlx3* are highly expressed in the posterior lobes but not in the flocculonodular lobe^42,43^ (Fig. 3e), challenging the evolutionary conservation of spatially specific gene expression patterns. Surprisingly, gene cohorts related to the distribution along A-P axis discovered in human showed almost no correlation with mouse data and vice versa, suggesting a dramatic cross-species difference (Fig. 3f; Supplementary Fig. S3b–d). For example, *PARM1* was enriched in the posterior lobes of human while in the anterior lobes of mouse, exhibiting completely reverse distribution across species (Fig. 3d, e). For the first time such a significant difference was uncovered and the functional interpretation remains to be explored.

Taking advantage of the integrated scRNA-seq and scATAC-seq data, GRNs involved in the A-P distribution of GCs were generated (Supplementary Fig. S3e). Several transcriptional factors seemed to be core regulators during the diversification, such as RORB, RORA, ESRRG in the anterior region and TLX3, ETV5, GLI3 in the posterior region (Fig. 3g). According to the integrated data, GCs in different regions showed preference of accessibility within several marker gene loci, consistent with their expression patterns (Fig. 3g). These genomic regions might be the specific enhancers modulating the region-specific activation of the gene expression in the developing human cerebellum.

Besides the separation along A-P axis, variance in dorsal-medial (DM) and ventral-lateral (VL) axis was also identified in the oEGL of GW13 spatial transcriptomic data. GC progenitors could be divided into four groups according to the expression of *EBF2*, *PRR35*, *GALNTL6*, and *HEY1* in the scRNA-seq data (Fig. 3h). *PRR35* was specifically expressed in the VL region of the oEGL, while *EBF2* and *HEY1* seemed to be enriched in the DM region (Fig. 3h; Supplementary Fig. S3f). The DM-VL difference reminded us of the heterogeneity between cerebellar vermis and hemispheres. *EN1* plays a vital role in the development of cerebellar vermis^44^ and it was highly expressed in most of the anterior *EBF2*^+^ subtype in our data (Fig. 3h). We speculated that *PRR35* and *EBF2* were uniquely expressed in the GC progenitors at hemisphere and vermis, respectively. *GALNTL6* was enriched in the flocculonodular lobe as reported^21^. The *HEY1*^*+*^ cells were located in the most posterior region and they might develop into the nodule of the cerebellum.

Additional spatial patterns were also detected, which seemed not to exactly overlap with the characteristics mentioned above. Both *NPY* and *PPP1R17* were expressed in the *GALNTL6*^+^ GC progenitor subtype, while *NPY* was also detected in all *PRR35*^+^ cells and *PPP1R17* was only detected in part of them (Supplementary Fig. S3g). The biological importance of multidimensional patterns in GC lineages awaits future investigation. To understand the functional implication of various GC sublineages, we referred to the Human Phenotype Ontology database^45^ to inspect the risk genes associated with the cerebellar diseases. Acrocallosal syndrome featured with cerebellar hypoplasia is associated with *GLI3* mutation. *GLI3* was highly expressed in the flocculonodular lobe GC progenitors (Fig. 3d), suggesting the important role of specific lobule in the cerebellar development.

In conclusion, we dissected the spatial distribution pattern of genes in the developing human cerebellum along the A-P axis or DM-VL axis. Significant differences in the spatial gene expression pattern along A-P axis between human and mouse was discovered.

### UBC lineage development

**Fig. 4:**
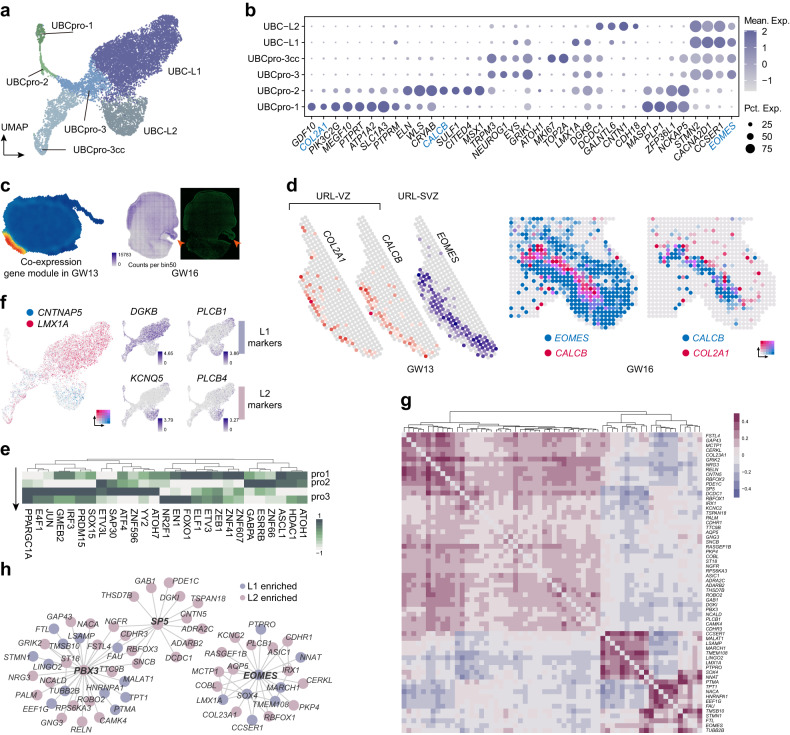
UBC progenitors and GRN of UBC sublineages. **a** Visualization of the UBC lineages. UBCpro-3cc was short for S/G2/M phase UBCpro-3. **b** Dot plot showing the expression patterns of marker genes of different cell types in **a**. Genes used in the following illustration of the compartmentation of the RL are colored bule. **c** Heatmap showing the high strength of co-expressed gene module containing *EOMES* and *CALCB* in URL (left). The RNA count map and fluorescence image of human GW16 cerebellum with URL marked by arrowheads (right). **d** Spatial gene expression patterns showing the distribution of *COL2A1*^*+*^ cells in URL, while *CALCB*^*+*^ and *EOMES*^*+*^ cells established the VZ and SVZ of URL in both GW13 and GW16 human cerebellums. **e** Heatmap showing the scaled regulon strength in different UBC progenitors. **f** Gene expression patterns of *CNTNAP5* and *LMX1A* in different UBC sublineages (left). Gene expression patterns of the reported markers for UBC sublineages (right). **g** Heatmap showing the correlation coefficients of marker genes for UBC sublineages. **h** GRN showing the potential regulatory programs of different UBC sublineages.

The UBCs are excitatory interneurons enriched in the median cerebellar cortex and part of flocculus/paraflocculus complex^13^. Both UBCs and GCs are considered as neuronal lineages generated from *WLS*^+^ progenitors in the RL^46^. Here, we described a newly characterized potential neuroepithelium (labeled as UBCpro-1) which may give rise to classic *WLS*^*+*^*CALCB*^+^ UBC progenitors (labeled as UBCpro-2) (Fig. 4a).

According to the scRNA-seq data, we found a new cluster of *COL2A1*^*+*^*PIK3C2G*^*+*^ progenitors (UBCpro-1) which expressed high level of stemness-related markers such as *SOX2* and *CYP26B1* (Fig. 4b; Supplementary Fig. S4a). According to the UMAP embeddings, there seemed to be some relevance between UBCpro-1 and classic *WLS*^+^ UBC progenitors (Fig. 4a). We then referred to the spatial transcriptomic data to investigate the distribution of these cells. Firstly, the RL was defined by co-expressed gene module analysis of GW13 data and by the morphology of GW16 data (Fig. 4c; Supplementary Fig. S4b). UBC markers including *EOMES*, *LMX1A* and *OTX2* were detected in the labeled RL regions^47^ (Fig. 1i, k; Supplementary Fig. S4c). The compartmentation of the RL^VZ^ and the RL^SVZ^ (subventricular zone of the RL), which was featured by the expression of *CALCB* and *EOMES*, respectively^47^, was also recaptured in our spatial transcriptomic data (Fig. 4d). The inside-out structure of *CALCB* and *EOMES* was observed and *COL2A1* seemed to be highly expressed in the most inside region (Fig. 4d). Thus, we speculated that the UBCpro-1 was the neuroepithelium which gave raise to classic *WLS*^*+*^*CALCB*^+^ UBC progenitors. Regulon-based analysis was also applied and identified key transcription factors, such as ASCL1, ETV3, ELF1 and ATOH1, which may regulate the cell state transition during differentiation of UBC lineages (Fig. 4e).

Mature UBCs could be divided into two subtypes based on the expression of *CALB2* or *GRM1*^13,48,49^. We also identified two potential subtypes of UBCs which could be distinguished by the expression of *LMX1A* and *CNTNAP5* (Fig. 4f). *LMX1A*^+^ UBCs were more likely to be the typical *GRM1*^+^ UBCs with molecular features as reported previously^50^ and *CNTNAP5*^+^ UBCs were possibly immature *CALB2*^+^ UBCs (Fig. 4f). KEGG enrichment analysis indicated that genes highly expressed in the *CNTNAP5*^+^ cells, such as *KCNQ5* and *PLCB1*, were associated with cholinergic synapse (Supplementary Fig. S4d) which was found to be functionally related to the *CALB2*^+^ UBCs^13^. Using markers of two UBC lineages (Fig. 4g), GRNs involved in the diversification of UBC lineages were constructed (Fig. 4h). We found EOMES and PBX3 as core regulators controlling the specification of all UBC lineages, while SP5 might be critical for the development of *CNTNAP5*^+^ sublineage (Fig. 4h). Integrated analysis of human and mouse scRNA-seq datasets^19^ identified a very similar expression pattern of *SP5* across species, indicating the conserved role of SP5 in regulating the diversification of UBC lineages (Supplementary Fig. S4e). Altogether, our analyses revealed the potential developmental progress of UBC lineages and the underlying gene regulatory logic likely involved in the early cell fate specification.

### VZ progenitors and interneuron development

**Fig. 5:**
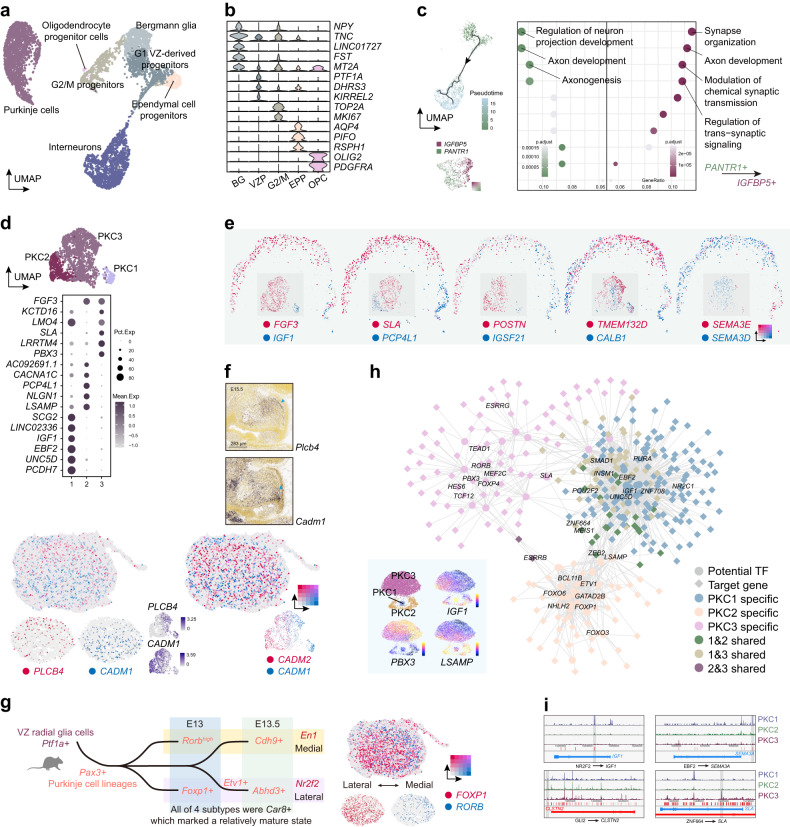
Trajectory of interneuron lineages and PKC subtypes. **a** Visualization of different progenitors, GABAergic neurons and glia cells. **b** Violin plots showing the expression patterns of marker genes for cell types in **a**. **c** Pseudotime trajectory from the VZ progenitors to interneurons (left top). Gene expression patterns of *PANTR1* and *IGFBP5* (left bottom). GO enrichment results of *PANTR1*^*+*^ and *IGFBP5*^*+*^ interneurons (right). **d** Visualization of three PKC subtypes (top) and dot plot showing corresponding expression patterns of marker genes (bottom). **e** Multidimensional spatial gene expression patterns of PKCs revealed by GW16 Stereo-seq and corresponding gene expression patterns revealed by scRNA-seq. **f** Gene expression patterns of *PLCB4*, *CADM1* and *CADM2* in PKCs revealed by the scRNA-seq and GW13 Stereo-seq (bottom). In situ hybridization results of *Plcb4* and *Cadm1* in mouse at E15.5 (top) adopted from Allen Brain (https://developingmouse.brain-map.org). **g** Schematic overview of PKC developmental progress in mouse (left). Expression of *FOXP1* and *RORB* in human GW13 cerebellum along L-M axis (right). **h** GRN showing potential regulatory programs of different PKC subtypes (top right). Visualization of unsupervised clusters in scATAC-seq data and scores of their marker genes with blue representing low levels and yellow representing high levels (bottom left). **i** Visualization of accessible regions of different genes in three PKC subtypes. Specific accessible regions are emphasized by deep grey panel. Blue or red color of gene loci represents the reverse or forward transcriptional direction, respectively.

Similar to the role of ganglion eminence in the developing forebrain, the VZ gives rise to multiple types of GABAergic neurons including PKCs and various interneurons^8^. To characterize the development of GABAergic lineages, VZ-derived progenitors and neurons were selected for further analysis (Fig. 5a). Despite the neuronal lineages, we discovered several non-neuronal cells including *OLIG2*^+^*PDGFRA*^+^ oligodendrocyte progenitor cells and *PIFO*^+^*RSPH1*^+^ ependymal cell progenitors^51,52^ (Fig. 5a, b). Bergmann glia cells which facilitate the migration of PKCs and GCs could also be identified according to the expression of *TNC* and *FAM107A*. Moreover, *LINC01727* seemed to be a specific marker of Bergmann glia in the cerebellum (Fig. 5b). Interestingly, the major exons in *LINC01727* gene are truncated in rodent^53^ (Supplementary Fig. S5a), suggesting the potential cross-species difference.

Since the neurogenesis of PKCs has already been completed in our samples, pseudotime analysis was only applied to the potential interneuron lineages (Fig. 5c; Supplementary Fig. S5b). We noticed that the postmitotic interneurons could be dissected through mutually exclusive expression pattern of *PANTR1* and *IGFBP5*. A recent study indicated that *IGFBP5* was a pan-marker of Golgi interneurons during human embryonic cerebellar development^54^. GO enrichment analysis suggested the shift from active axonogenesis in *PANTR1*^+^ cells to active synapse organization in *IGFBP5*^+^ cells (Fig. 5c), indicating the maturation progress of the interneurons.

### PKC subtypes and their GRNs

PKCs execute critical functions in the cerebellar circuits with a relatively limited cell number^55^. Mature PKCs could be divided into two subtypes by the alternating strips of Zebrin II (protein encoded by gene *ALDOC*) expression along the lateral-medial (L-M) axis^56^. Here we identified three molecularly distinct subtypes of PKCs labeled as PKC1, PKC2 and PKC3 in human (Fig. 5d; Supplementary Fig. S5c). PKC1 was *EBF2*^+^*IGF1*^+^ cells, corresponding to the reported Zebrin II negative PKCs^57–59^ (Fig. 5d). Therefore, both *LSAMP*^*+*^*PCP4L1*^*+*^ PKC2 and *SLA*^*+*^*PBX3*^*+*^ PKC3 with the expression of *FGF3* might be subtypes of Zebrin II positive PKCs (Fig. 5d). Separation of both *IGF1*-*FGF3* and *PCP4L1*-*SLA* could be found in our GW16 spatial transcriptomic data (Fig. 5e), suggesting the correspondence between three molecular subtypes of PKCs and their spatial organizations. Furthermore, PKCs could also be dissected multidimensionally (Fig. 5e). The distribution of *POSTN* and *IGSF21* resembled the A-P pattern mentioned in the GC lineages (Fig. 5e). While POSTN was reported to be a neurite outgrowth-promoting factor^60^, IGSF21 was shown to regulate inhibitory presynaptic differentiation^61^. Such a difference might implicate the functional diversification or the asynchronized developmental rates between anterior and posterior PKCs. Different members of semaphorin family proteins which regulated cell migration were found to be enriched in different regions (Fig. 5e). Previous study showed that Sema3e and Sema3d mediates endothelial cell repulsion through distinct molecular pathways^62^. Similar mechanisms may underpin the spatial organization of PKC subtypes.

We also discovered a conserved separation between *CADM1* and *PLCB4/CADM2* along the dorsal-ventral (D-V) axis, especially in the posterior region during the early development of PKCs (Fig. 5f). The expression of *CADM1* seemed to be enriched in the ventral region, which could be detected in both human (GW13) and mouse (E15.5) (Fig. 5f). Such separation became relatively subtle later at GW16 and did not follow the distribution along D-V axis. Both CADM1 and CADM2 serve as membrane proteins engaged in the cell–cell adhesion and are important for the neurite arborization^63,64^. *CADM1* was reported as an autism-associated gene and its knockout would lead to smaller cerebellum and decreased synapse of PKCs^63^. Our discovery suggested the potential role of *CADM1* in the organization of PKCs during the early development and the disturbance of this distribution may also underpin the autism caused by *CADM1* mutation.

We also inspected the cross-species similarity of PKCs (Supplementary Fig. S5d–i). As reported in a recent study, mouse *Car8*^+^ PKCs could be grouped into four subtypes^26^ (Fig. 5g). Spatial segregation of *FOXP1*^high^ and *RORB*^+^ PKCs along L-M axis was observed in GW13 spatial transcriptomic data, which was similar between human and mouse (Fig. 5g). However, both *ABHD3* (or *ETV1*) and *CDH9* which marked the proposed late-born PKCs were hardly detected in GW13 spatial transcriptomic data (Supplementary Fig. S5j). It remains unclear whether such difference was due to the immature state of late-born PKCs or the cross-species heterogeneity.

Taking advantage of integrated scRNA-seq and scATAC-seq data, we also generated the GRNs and investigated the potential regulatory elements involved in the diversification of PKC lineages (Fig. 5h). PKCs could also be classified into three types in our scATAC-seq data (Fig. 5h). We found that EBF2 might specifically activate *SEMA3A* through an enhancer distant from the promoter (Fig. 5i), which was consistent with the shapely decreased *Sema3a*^+^ PKCs in *Ebf2*-null mice as reported^65^. NR2F2 and ZNF664 might also be involved in the differentiation of different PKC subtypes (Fig. 5i).

## Discussion

In this study, we employed single-cell multi-omic technologies to systematically investigate the early development of the embryonic human cerebellum. Acquisition of high-quality data facilitated the in-depth understanding of molecular heterogeneity, spatial organization, and developmental trajectories of neuronal lineages in the developing human cerebellum. Based on the integration of scRNA-seq and scATAC-seq, we characterized the GRNs involved in the development of GC lineages. Conserved GC progenitors exhibiting varying degrees of stemness were identified. Beyond stemness, GC lineages could be further distinguished by their spatial characteristics which was validated in our spatial transcriptomics data. Three GC sublineages were matched to the different lobes of the cerebellum. Cross-species heterogeneities of gene cohorts enriched in either anterior (*BARHL1*^+^) or posterior (*TLX3*^+^) regions were identified, represented by *PARM1* with completely reverse expression pattern along A-P axis in humans and mouse. Potential regulators involved in the diversification of anterior and posterior GCs were identified based on GRNs. GC progenitors could also be dissected according to the expression of *EBF2*, *PRR35*, *GALNTL6,* and *HEY1*, suggesting the complexity of the spatial organization in GC lineages during the development. Our analysis suggested the potential *COL2A1*^+^ neuroepithelium of UBC lineages. PKC subtypes could be dissected multidimensionally in our spatial transcriptomics data. Different semaphorin proteins were enriched in different regions, suggesting the distinct molecular pathways involved in the organization of PKC subtypes. Potential regulators of each PKC subtype were also identified.

Morphology analysis of cerebellum has revealed cross-species differences in the development of URL^1^. Our investigation brought forth additional insights into the aspect of molecular features. Although the general cellular architecture of the developing cerebellum is largely conserved, many differences seem to exist in the gene expression patterns and spatial distribution of certain cell types between humans and mice. For example, the AT+GCPs and ND+GCPs were identified in both species, while human GC progenitors were featured by high expression levels of *MGP* and *NPY* (Supplementary Fig. S2h). These genes were almost undetectable in mouse GC progenitors^15,19^, which could be validated in another dataset^26^. Such cross-species differences are unlikely to be attributed to the data quality, given that the extremely high expression levels of *MGP* and *NPY* were detected in human. Furthermore, though *HEY1* was suggested as a critical regulator of GC lineage development^27^, different expression patterns of *HEY1* could be observed between human and mouse. *Hey1* was almost co-expressed with *Atoh1* in mouse, while it was confined in a small fraction of human *ATOH1*^+^ GC progenitors (Supplementary Fig. S2a, f). Whether these differences in progenitors account for the different sizes of human and mouse cerebellums remains to be determined. Since the cerebellar development continue to postnatal 14 months in human and postnatal 20 days in mouse, more detailed temporal analyses covering the neonatal stages are needed to fully reveal the developmental landscapes in different species.

Cross-species differences were also observed concerning the region-specific gene expression patterns. Previous works suggested that several genes might be involved in spatial organization among cells in the cerebellum^14^. Here we further characterized the distinct gene cohorts in human and mouse GC lineages. We speculate that similar systems comprising various gene modules participate in spatial organization of GCs in both species, but the genes involved are not identical. Different gene modules influence cell distribution in different dimensions. Due to the intersection of these gene modules, unsupervised classification results may not represent genuine biological ontologies. More spatial data covering different species and other experiments are needed to validate this hypothesis. It remains unclear how GC progenitors obtain their spatial identity and whether these identities are intrinsically determined or changed during the tangential migration. Previous studies have shown a transient alternative distribution of *Ebf2* in mouse GC progenitors, mimicking the distribution of Zebrin II negative PKCs^59^. Since we also found the similar patten along A-P axis between *BARHL1-TLX3* in GCs and *POSTN-IGSF21* in PKCs, the potential cross-talk between GCs and PKCs remains intriguing to be investigated. Furthermore, it was reported that the lobes in the posterior cerebellar hemisphere, which tend to be activated by cognitive tasks, occupy larger size in human than in other non-human primates and rodents^66,67^. Since *PARM1* was found to be an oncogene which can promote proliferation^68,69^, the possibility that *PARM1* with reverse expression pattern promotes the enlargement of posterior cerebellar hemisphere in humans warrants further investigation.

Besides the gene expression patterns revealed by scRNA-seq, we also characterized the GRNs involved in the diversification of GCs along A-P axis based on the integration of scRNA-seq and scATAC-seq. However, it should be noticed that the sequencing depth achieved in our study are still insufficient to fully characterize the regional heterogeneity of GCs. Integration of large-scale Smart-seq data and scATAC-seq data would provide more insights into the diversification of GC lineages. During the lineage development analysis of the RL-derived neurons, we did not identify the *Lhx9*^+^*Pou3f2*^+^ interposed nucleus neurons or *Tbr1*^+^ fastigial nucleus neurons^70,71^. It may result from the bias during the sampling. Additionally, how the GC progenitors are generated from the RL remains uncertain. VZ-derived PKCs were generally characterized into three subtypes and the multidimensional features were described. Though PKCs in mouse could be grouped into four subtypes^26^ (Fig. 5g), more molecular subtypes could be classified (Supplementary Fig. S5i). Whether these subtypes represent functionally or spatially distinct PKCs or the consequence of over-clustering requires experimental validation.

In summary, our study generated a large-scale multi-omics atlas of the developing human fetal cerebellum. This comprehensive atlas sheds light on the molecular mechanisms underlying early human cerebellar development and evolution. We foresee that the key regulators identified in this study will be leveraged in vitro to generate desired cerebellar cells for future clinical applications. This dataset will also be important to enhance our understanding of the linkage between molecular variation and cell types in neurodevelopmental disorders.

## Materials and methods

### Human sample collection

The human clinical tissues of pregnancies at GW13–18 were obtained upon therapeutic termination of pregnancy at Obstetrics and Gynecology Hospital of Fudan University. An informed consent document was signed by the patient before collection of the human sample. The whole experiment was examined by Ethics Committee of Obstetrics and Gynecology Hospital of Fudan University (2020-157).

### Brain tissue dissection and cell dissociation

The human cerebellar tissues were dissected in ice-cold normal saline under the dissection microscope. The cerebellums from all samples were collected. Half of the human cerebellum was stored in liquid nitrogen for scATAC-seq. Each tissue for scRNA-seq was dissociated in 500 μL dissociation agent (400 U/mL DNaseI on hibernate E buffer, 10 U/mL papain) at 37 °C on a thermocycler for 15 min. Dissociation was terminated by 500 μL of 10% FBS in Hibernate E buffer. Cells were centrifuged at 4 °C for 5–10 min. After removal of the supernatant, 1 mL HA buffer was added and cells were blown up with straws repetitively to generate monoplast suspension.

### scRNA-seq

#### Cell preparation

For the quality check and counting of single cell suspension, the cell survival rate was generally > 80%. The cells that passed the test were washed and resuspended to a suitable cell concentration of 700–1200 cells/μL for 10X Genomics ChromiumTM. The system was operated on the machine.

#### Gel bead in emulsion (GEM) creation and thermal cycling

GEMs were constructed for single-cell separation according to the number of cells to be harvested. After GEMs were normally formed, GEMs were collected for reverse transcription in a PCR machine for labeling.

#### Post cycling cleanup and cDNA amplification

The GEMs were oil-treated, and the amplified cDNA was purified by magnetic beads, and then subjected to cDNA amplification and quality inspection.

#### Library preparation and quantification

The 3ʹ gene expression library was constructed with the quality-qualified cDNA. After fragmentation, adaptor ligation, sample index PCR, etc., the library was finally quantitatively examined.

#### Sequencing

Cells were loaded onto the 10X Chromium Single Cell Platform (10X Genomics) at a concentration of 1000 cells/μL (Single Cell 3′ library and Gel Bead Kit v3) as described in the manufacturer’s protocol. Generation of GEMs, barcoding, GEM-RT cleanup, complementary DNA amplification and library construction were all performed as per the manufacturer’s protocol. Qubit was used for library quantification before pooling. The final library pool was sequenced on the Illumina Nova6000 instrument using 150-bp paired-end reads.

### Single-cell gene expression quantification

After single-cell sequencing, CellRanger (v7.0.0) was applied to generate the fastq format data, and perform the quality control and read counting of genes with default parameters. Human GRCh38 (hg38) reference genome was chosen to perform the genomic alignment of reads. Seurat (v4.3.0) was used to analyze the gene-cell data matrix. MiQC (v1.1.3)^72^ was adopted to remove low-quality cells (posterior.cutoff = 0.95, model.slot = “flexmix_model”, model.type = “spline”). The remaining low-quality cells or potential doublets were directly discarded (mitochondrial genes > 20% or features < 800 or features > 6500). DoubletFinder (v2.0.3)^73^ was then applied and predicted doublets were further removed (parameters were based on cell counts of each data). Hemoglobin genes were removed. Standard Seurat pipeline was applied to each dataset (VariableFeatures = 3000, resolution = 1, PC depended). COSG (v0.9.0) and the function FindMarkers in Seurat, was used to calculate marker genes for each cluster. Cluster identities were manually labeled based on reported marker genes. Besides the neuronal lineages, microglia (*CX3CR1*^+^*TREM2*^*+*^), macrophage (*FOLR2*^+^*VISG4*^*+*^) and endothelial cells (*CLDN5*^+^*ITM2A*^*+*^) were identified. Detailed parameters are provided in our code, which is available on GitHub (https://github.com/NeuroXplorer-XuLab/Multi-omics-human-cerebellum).

### Integration of human scRNA-seq data

Non-neural lineage cells were discarded from each dataset before the integration. FindIntegrationAnchors and IntegrateData functions from Seurat were applied to the entire integration process. For the integration of all neural lineages, supervised integration of scRNA-seq data was applied based on major markers selected from each cell type. For the analysis of certain neural lineage, cells were first selected and separated based on time point. Unsupervised integration was then applied. Analysis of interneurons did not separate the datasets because of the low cell counts. Due to the low feature counts in UBC, lower number of anchor features was used.

### Mouse scRNA-seq analysis

GEO datasets were acquired from the GEO website (GSE118068^19^) and are publicly accessible at https://www.ncbi.nlm.nih.gov/geo. We mainly utilized data from E10, E12, E14, E16, E18, P0, P5 and P7. Other datasets of E13–18 were collected from PRJEB23051^15^ in European Nucleotide Archive. Cells expressing < 800 genes were removed. Standard Seurat pipeline was then applied to each data. Similar unsupervised integration was applied. Pearson correlation coefficient was calculated within each integrated data by cor.test provide by R (v4.2.2). Mouse excitatory lineage cells, as well as GABAergic neurons, from each time point were selected and integrated. *Rtn1* was used as the marker of neuronal lineages as well as astrocytes. Different kinds of excitatory neurons were dissected by time periods and then integrated within each lineage for further analysis as shown in Supplementary figures. Integration local inversed Simpson’s index (LISI) was calculated via R package lisi (v1.0)^74^.

### Cell cycle analysis

The Seurat package provided cell cycle-related genes, comprising 43 genes associated with the S phase and 54 genes linked to the G2/M phase (Supplementary Table S7), which were utilized to determine each cell’s stage by executing the CellCycleScoring function within Seurat.

### GO enrichment, KEGG pathway, and GSEA analyses

The clusterProfiler package (v4.7.1)^75^ was employed to discern enriched GO terms and KEGG pathways associated with marker genes. Marker genes with a *P*-value < 0.05 from prior studies were selected and the enrichGO and enrichKEGG functions were utilized (ont = “BP”, qvalueCutoff = 0.05, pAjustMethod = “BH”). Moreover, the gseGO and gseKEGG functions were implemented to further investigate potential GO and KEGG pathways, using genes calculated via the FindMarker function in Seurat (logfc.threshold = 0 and min.diff.pct = 0).

### Predict stemness with CytoTRACE and StemSC

Count data extracted from each dataset was input into the CytoTRACE (v0.3.3) and StemSC (v1.0.1). Subsequently, CytoTRACE and StemSC scores were employed to visualize the stemness of each cell type and to facilitate the construction of pseudotime trajectory in Monocle3.

### Trajectory analysis

Splicing-specific count data were computed using Velocyto (v0.17.17)^76^ for RNA velocity analysis, employing default parameters. The generated loom files were further analyzed with scVelo (v0.2.3)^77^. Gene selection, normalization and moment estimation as well as RNA velocity estimation were performed (min_shared_counts=30, n_top_genes=2000 and n_pcs=30, n_neighbors=30). RNA velocities were visualized on UMAP using the stream embedding function. Monocle3 was also utilized to infer pseudotime trajectories for the major cell types. A Monocle cell dataset was constructed using data calculated by Seurat.

### Nucleus isolation from frozen brain tissue

Frozen human tissue was cut into small pieces and ground in 2 mL of ice-cold homogenization buffer (20 mM Tris, pH 8.0 (Thermo Fisher Scientific), 500 mM sucrose (Sigma), 50 mM KCl (Thermo Fisher Scientific), 10 mM MgCl_2_ (Thermo Fisher Scientific), 0.1% NP-40 (Roche), 1× protease inhibitor cocktail (Roche), and 1% nuclease-free BSA, and 0.1 mM DTT). To release the nuclei, tissues were homogenized by strokes. A total of 30 μm cell strainer was used to filter nuclei into a 15 mL centrifuge tube. After centrifugation for 5 min at 4 °C, nuclei were obtained and washed twice with 1 mL of ice-cold blocking buffer (1× PBS supplemented with 1% BSA). After another centrifugation, the nuclei were collected in 50 μL of 1× PBS containing 1% BSA and counted with DAPI.

### scATAC-seq library preparation and sequencing

We used DNBelab C Series Single-Cell ATAC Library Prep Set (MGI, #1000021878), to prepare scATAC-seq libraries. The transposed single-nucleus suspensions were converted to barcoded scATAC-seq libraries. After procedures including droplet encapsulation, pre-amplification, emulsion breakage, capture bead collection, DNA amplification and purification, indexed sequencing libraries were prepared according to the user guide. We measured the concentrations of sequencing library with Qubit ssDNA Assay Kit (Thermo Fisher Scientific). The library was sequenced using a paired-end 50 sequencing scheme by the BGISEQ-500 platform at China National GeneBank^78^.

### scATAC-seq data processing and construction of GRNs

Raw sequencing reads from the BGISEQ-500 sequencer were filtered, demultiplexed using PISA, and aligned to the hg19 human genome. Fragmented data was further processed with ArchR (v2.0.1)^31^. Cells with transcription start site enrichment scores below 4 and fragment numbers less than 1000 were removed. Doublet analysis was performed using the addDoubletScores and filterDoublets functions in ArchR. “Iterative LSI” was then executed, utilizing major pc2-pc30 to cluster with Seurat’s FindClusters function at a resolution of 0.1. Gene activity scores were employed to identify distinct cell types based on various marker genes. Clusters were validated using several well-established marker genes as previously described. A total of 227,532 peaks were identified via peak calling using MACS2 (v2.1.1), and peaks were linked to genes by the addPeak2GeneLinks function.

The construction and analysis of GRNs in our study involved a three-step process: (1) examination of snATAC-seq and snRNA-seq datasets; (2) integration of these datasets; and (3) inference of *cis*-regulatory interactions to define a transcription factor-gene GRN (TF-gene GRN). In terms of single-cell transcriptomic and epigenomic data integration, we employed the “addGeneIntegrationMatrix” function to incorporate the gene expression matrix of snRNA-seq data onto the “geneScoreMatrix” of snATAC-seq data in both wild-type and mutant mice.

The method utilized for the subsequent definition of TF-gene GRN, involving the inference of *cis*-regulatory interactions, is complex and a separate manuscript is currently being prepared in the laboratory of Dr. Qiang Tu. The insights gained from GRNs of our study serve as preliminary findings and may provide potential directions for future hypothesis generation rather than as definitive conclusions. GRN results were visualized by Cytoscape^79^.

### Identification of marker genes among clusters in scATAC-seq

We use getMarkerFeatures function (useMatrix = “GeneScoreMatrix”, bias = c (“TSSEnrichment”, “log_10_ (nFrags)”, testMethod = “Wilcoxon”)) and FDR ≤ 0.01 and |log_2_FC | ≥ 1 to find marker genes among clusters in scATAC-seq.

### Stereo-seq library preparation and sequencing

Stereo-seq library preparation and sequencing were adapted according to the standard protocol V1.1 with minor modifications^80^. Tissue sections were adhered to the Stereo-seq chip, and incubated in –20 °C methanol for 30 min fixation, followed by nucleic acid dye staining (Thermo Fisher, Q10212) and imaging (Ti-7 Nikon Eclipse microscope). For permeabilization, tissue sections were permeabilized at 37 °C for 5 min. The cDNA was purified using AMPure XP beads (Vazyme, N411-03). The indexed scRNA-seq libraries were constructed according to the manufacturer’s protocol. The sequencing libraries were quantified by Qubit ssDNA Assay Kit (Thermo Fisher Scientific, Q10212). DNBs were loaded into the patterned Nano arrays and sequenced on MGI DNBSEQ-Tx sequencer (50 bp for read 1, 100 bp for read 2).

### Raw Stereo-seq data processing

Fastq files were produced using an MGI DNBSEQ-Tx sequencer, with read 1 containing CID (1–25 bp) and MID (26–35 bp), while read 2 comprised the cDNA sequences. CID sequences on the first reads were initially mapped to the designed coordinates of the in situ captured chip obtained from the first round of sequencing, permitting a single base mismatch to account for sequencing and PCR errors. Reads with MIDs containing either N bases or over two bases with a quality score below 10 were discarded. CID and MID associated with each read were appended to the respective read headers. Retained reads were then aligned to the reference genome (hg19) using STAR^81^, and mapped reads with MAPQ > 10 were counted and annotated to their corresponding genes. UMIs with the same CID and gene locus were collapsed, allowing a single mismatch to correct for sequencing and PCR errors. Ultimately, this information was employed to generate a CID-containing expression profile matrix. The entire process was integrated into a publicly available pipeline named SAW, accessible at https://github.com/BGIResearch/SAW.

### Stereo-seq data analysis and clustering

X-Y coordinates and overall MID counts were acquired using the st.io.read_gef function with the parameter bin_size=50 in Stereopy (https://github.com/BGIResearch/stereopy), simultaneously preparing the data for subsequent analyses. Filter_cells function was applied to exclude low-quality cells with parameters min_gene=50 and min_n_genes_by_counts=3. The Hotspot package was employed for the identification of informative genes and gene modules. Standard Seurat pipeline was used for further data cleaning and analysis.

### Supplementary information

Supplementary Figures

Supplementary Tables

## Data Availability

The sequencing data have been uploaded to China National GeneBank DataBase (https://db.cngb.org/) with human fetal cerebellum data in project CNP0005078 and CNP0002781.
