# Supplementary material for: Single-cell multi-omics analysis of lineage development and spatial organization in the human fetal cerebellum
Source: Cell Discov. 2024 Feb 26;10:22. doi: 10.1038/s41421-024-00656-1 (PMC10897198; doi:10.1038/s41421-024-00656-1)
Supplement: Supplementary file 1 — Supplementary Figures [file 41421_2024_656_MOESM1_ESM.pdf]

## Supplementary Information

# Single-cell multi-omics analysis of lineage development and spatial organization in the human fetal cerebellum

Fuqiang Yang[#], Ziqi Zhao[#], Dan Zhang[#], Yu Xiong[#], Xinran Dong[#], Yuchen Wang, Min Yang, TaoTao Pa, Chuanyu Liu, Kaiyi Liu, Yifeng Lin, Yongjie Liu, Qiang Tu, Yashan Dang, Mingyang Xia[*], Da Mi[*], Wenhao Zhou[*], Zhiheng Xu[*]

**This file includes:**

Supplementary Figures S1-5

Figure legends

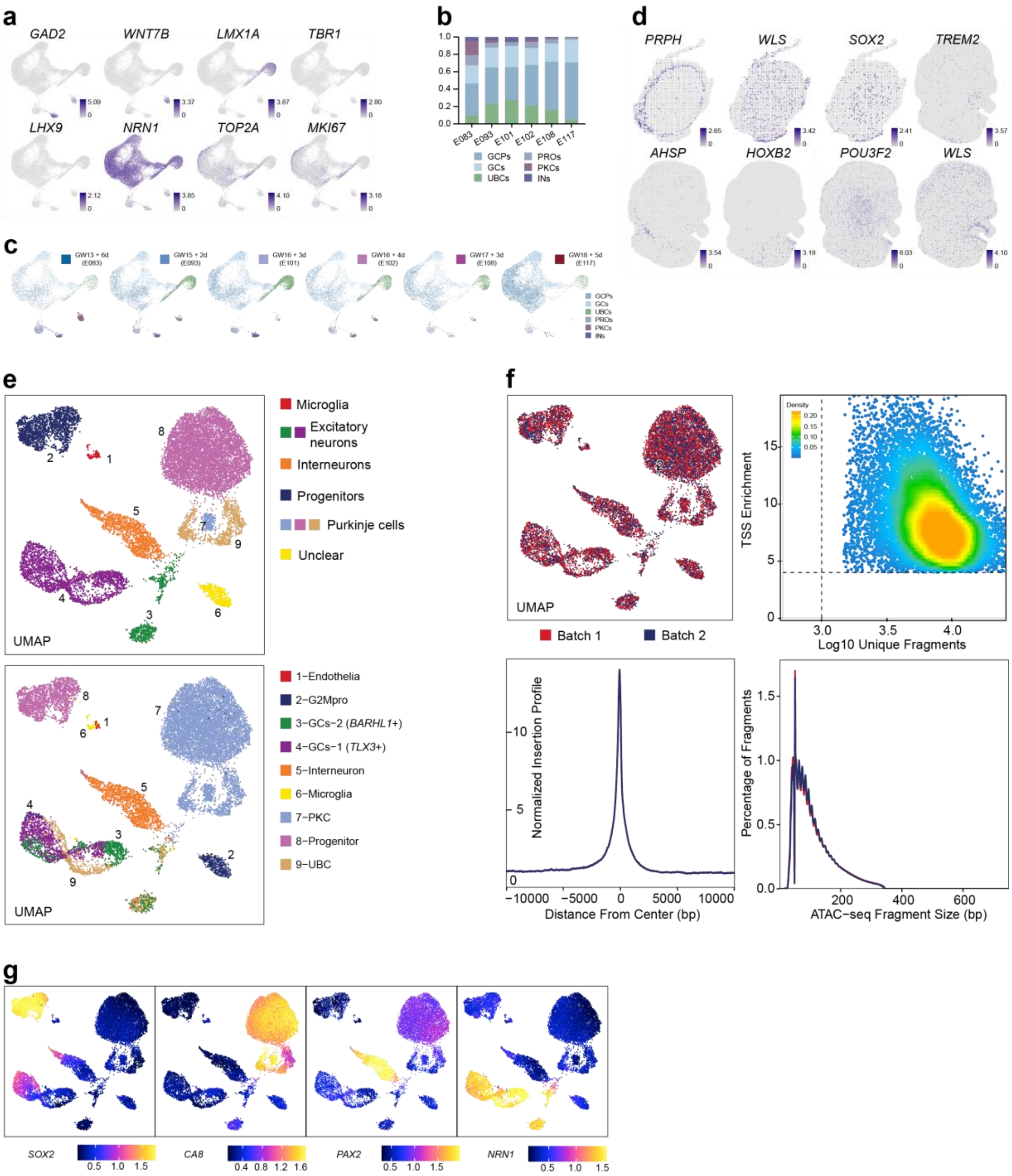


**Supplementary Fig. S1 Expression of marker genes and cell compositions in muti-omics data, related to Fig. 1. a** More gene expression patterns of marker genes. *GAD2* of interneurons, *WNT7B* of PKCs, *LMX1A* of UBCs, *TBR1 and LHX9* of deep cerebellar nuclei neurons, *NRN1* of excitatory neurons, *TOP2A* and *MKI67* of G2/M phase progenitors. **b** Bar plot showing the ratio of different neural lineage cells in each timepoint. **c** UMAP representations of cell composition in each timepoint. **d** More spatial gene expression patterns. **e** Visualization of scATAC-seq showing either unsupervised clustering result (top) or clustering result after the integration with scRNA-seq data (bottom). **f** Quality control results of scATAC-seq data. **g** Feature plots showing the gene score of marker genes of different cell types.

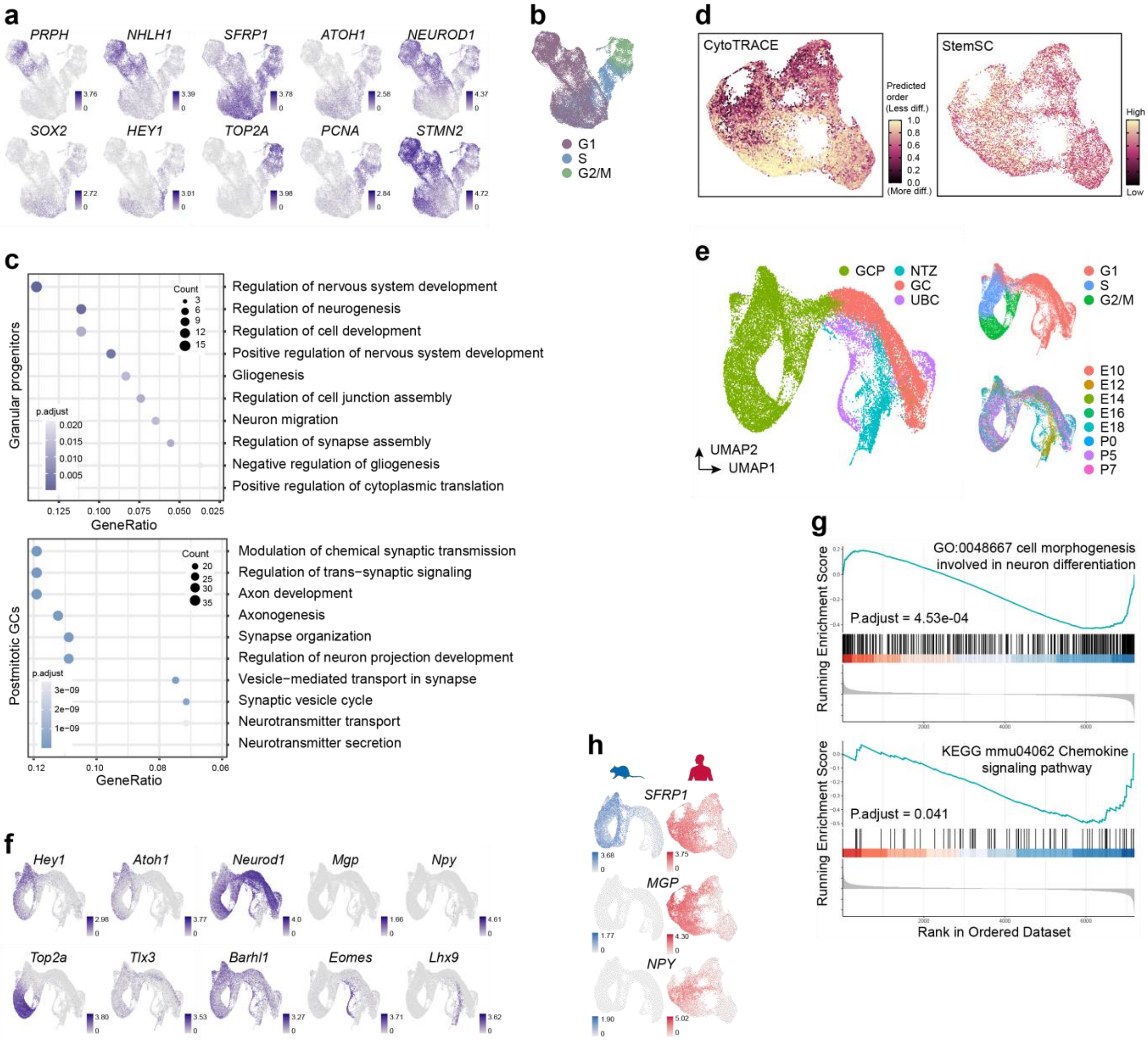


**Supplementary Fig. S2 Gene expression patterns of GC progenitors in human and mouse, related to Fig. 2. a** More gene expression patterns of marker genes in GC lineages. *PCNA*, G1 phase indicator. **b** Visualization of the cell cycle stage scoring result. **c** Dot plots of GO enrichment results based on marker genes of GC progenitors (top) or postmitotic GCs (bottom). **d** Visualization of the stemness inference results calculated by CytoTRACE (left) or StemSC (right). **e** Visualization of the excitatory lineages from mouse E10-P7 (GSE118068) (left). Cell cycle stage scoring result (right top) and distribution of cell lineages from different samples (right bottom). NTZ, nuclear transitory zone. **f** Gene expression patterns of marker genes of excitatory lineages in mouse. **g** GO and GSEA enrichment result of mouse AT+ GCPs compared with ND+GCPs, which was similar to Fig. 2e. **h** Different gene expression patterns of *MGP* and *NPY* in GC lineages between human and mouse. *SFRP1* was shared marker of GC progenitors.

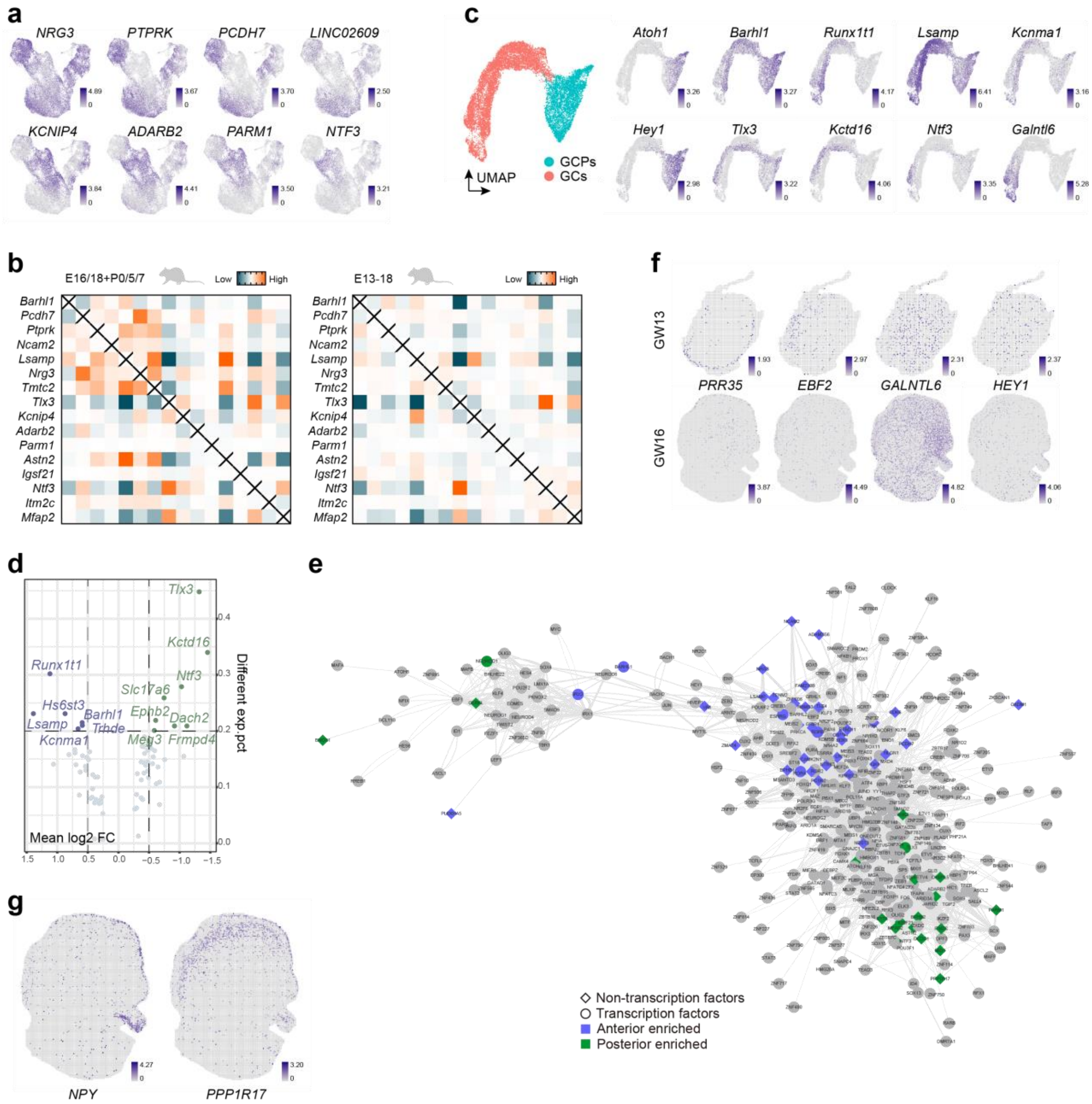

**Supplementary Fig. S3 Molecular and spatial diversity of GC sublineages, related to Fig. 3. a** More gene expression patterns of the anterior (top) and posterior (bottom) lobe specific genes in human. **b** Heatmap showing the correlation coefficients of A-P related genes in two published mouse scRNA-seq data, namely GSE118068 (left) and PRJEB23051 (right). **c** Visualization of the mouse G1 phase GC lineage cells from E16-P7 (GSE118068) (left). Expression patterns of potential spatial-related genes (right). *Hey1* and *Atoh1* marked GC progenitors. **d** Volcano plot showing the mouse specific gene cohorts which were proposed to be associated with anterior (blue) and posterior (green) distributions of GC lineage cells. **e** Spatial gene expression patterns of *PRR35*, *EBF2, GALNTL6, HEY1* in GW13 and GW16 human cerebellum. **f** Spatial gene expression patterns of *NPY* and *PPP1R17* in GW16. **g** Gene regulatory networks involved in the GCs distribution along A-P axis in human.

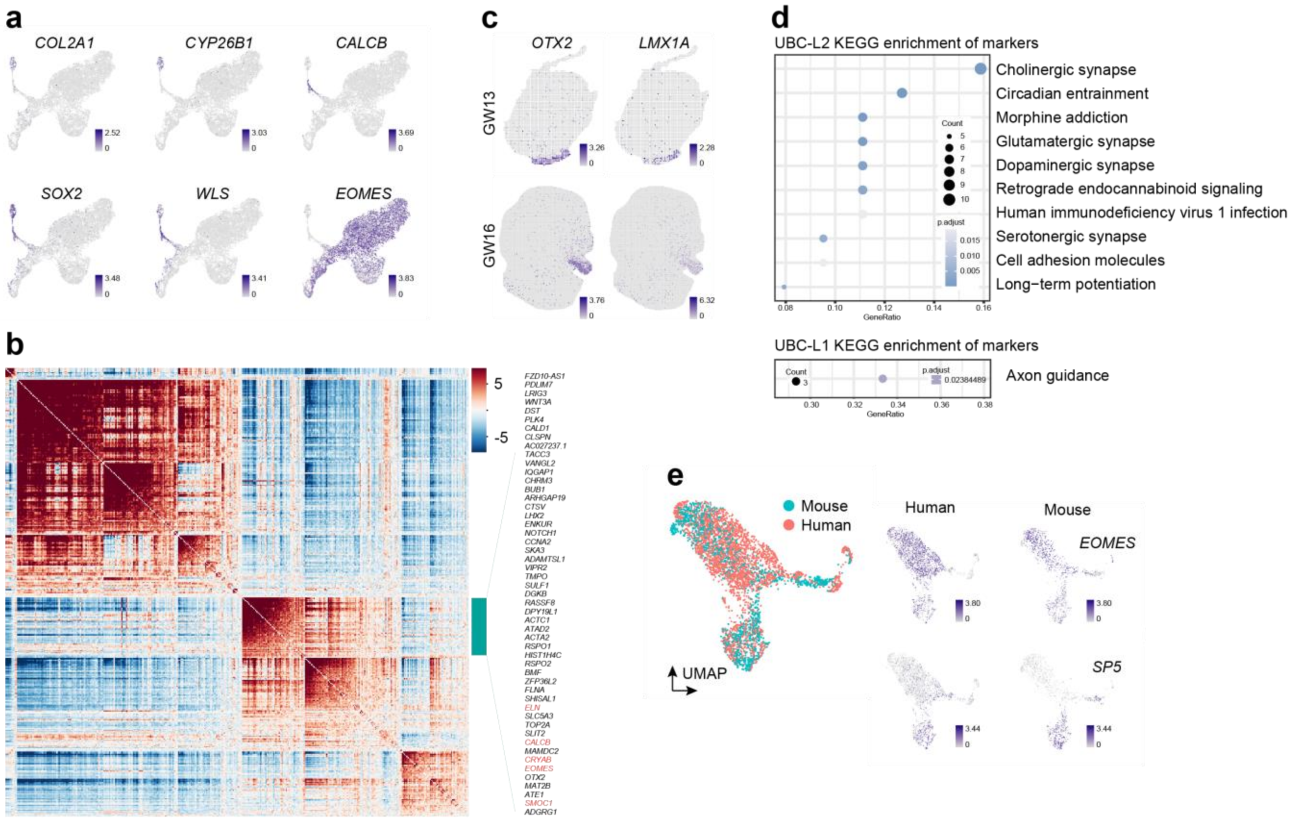


**Supplementary Fig. S4 UBC development in human and mouse, related to Fig. 4. a** More gene expression patterns of marker genes of UBC lineages. *COL2A1, CYP26B1, CALCB, SOX2, WLS* of progenitors. **b** Heatmap showing the gene correlation patterns in spatial RNA-seq data. Module including *EOMES* were marked in green and genes were listed. **c** Spatial gene expression patterns of *OTX2* and *LMX1A* in URL at GW13 (top) and GW16 (bottom). **d** Dot plots of KEGG enrichment results based on marker genes of UBC lineage 2 (top) or UBC lineage 1 (bottom). **e** Visualization of the integration results of mouse and human UBCs (left). Similar expression patterns of *Eomes* and *Sp5* could be observed (right).

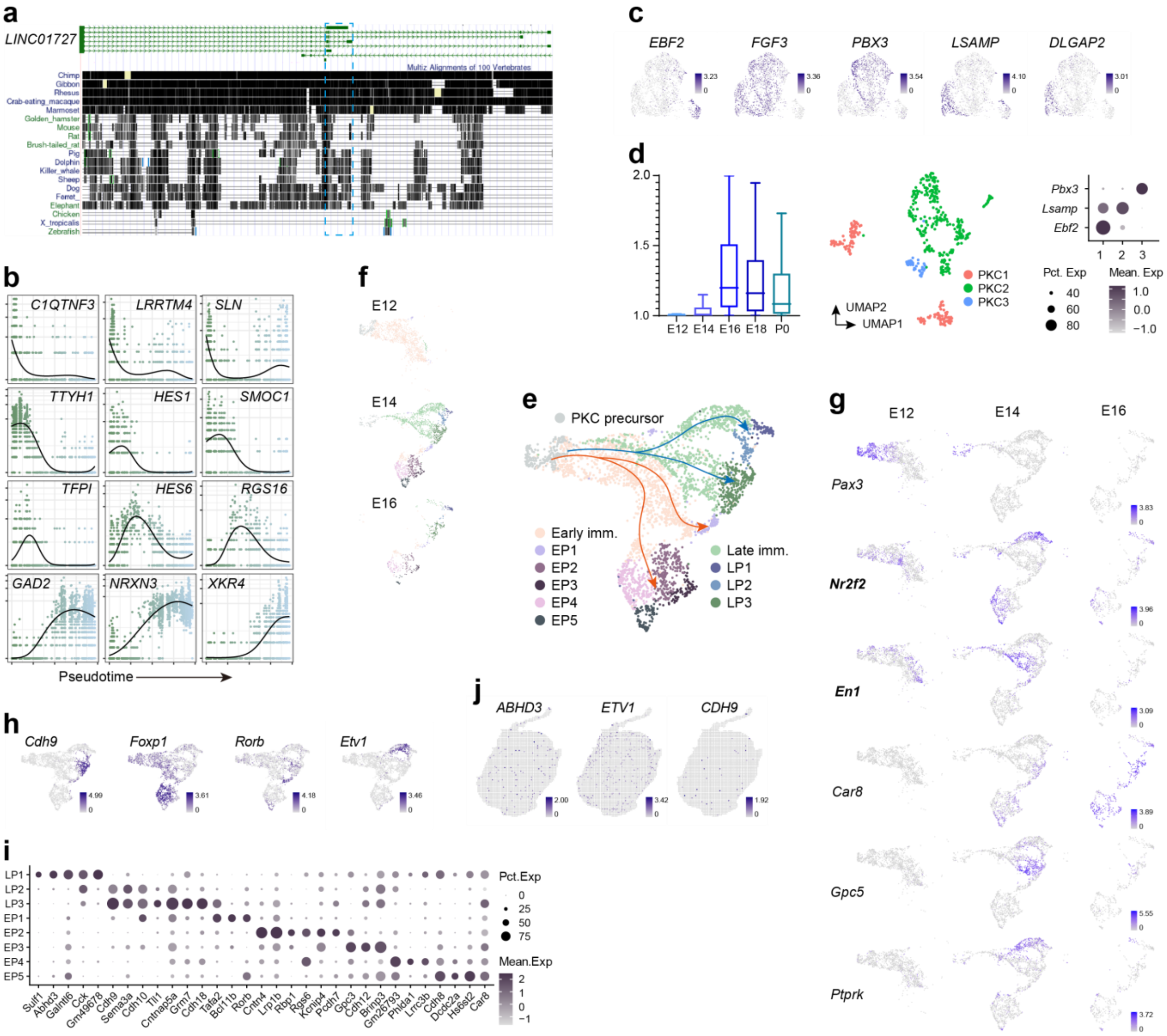

**Supplementary Fig. S5 GABAergic neuron development and diversity of PKCs in human and mouse cerebellum, related to Fig. 5. a** Snapshot of *LINC01727* genomic locus from UCSC browser (https://genome.ucsc.edu/) showing the cross-species difference. Main exons of *LINC01727* were emphasized by dash green panel. **b** Scatter plots and fitted curves showing the gene expression level changes of different genes over the psedotime during the development of interneurons. **c** Gene expression patterns of marker genes of different PKC subtypes. **d** Integration local inversed Simpson's index (LISI) between PKCs collected from human GW13 and mouse E12-P0 was calculated, which suggested a higher similarity between human GW13 PKCs and mouse E16 PKCs (left). Based on the expression of *Pbx3*, *Ebf2* and *Lsamp*, PKCs from mouse E16 could also be grouped into three subtypes (middle and right). **e-g** Visualization of the development of mouse PKCs (**e**). imm, immature PKCs. LP, late born PKCs. EP,

early born PKCs. Curves colored by red and bule indicted the different developmental stages and branches within certain colored lineage represented the potential two sublineages. The majority of red lineages were collected from E12 and the blue lineages were collected from E14 as shown in the cell composition of different timepoints (**f**). Almost no immature PKCs (i.e. *Car8* negative PKCs) could be detected in E16 (**f**). Temporal gene expression patterns (**g**) suggested *Pax3* as the marker of nascent postmitotic PKC precursors. *Nr2f2* and *En1* seemed to function in the both early and late born PKC lineages while they tended to be exclusively expressed, which may represent potential sublineages (**g**). *Car8* was selected to represent the relatively matured PKCs. Specific expression of *Gpc5* and *Ptprk* in late born immature PKCs could additionally distinguish them from the early born lineages (**g**). **h** Gene expression patterns of four genes proposed to be markers of PKC subtypes. *Etv1* was not detected in the *Car8*+ PKCs. **i** Dot plots showing the expression profiles of different *Car8*+ PKC subtypes in mouse marked in (**e**). **j** Spatial gene expression patterns of *ABHD1, ETV1* and *CDH9* in human GW13 cerebellum.
